# Impact of interleukin-21 in the pathogenesis of primary Sjogren's syndrome: increased serum levels of interleukin-21 and its expression in the labial salivary glands

**DOI:** 10.1186/ar3504

**Published:** 2011-10-26

**Authors:** Kwi Young Kang, Hyun-Ok Kim, Seung-Ki Kwok, Ji Hyeon Ju, Kyung-Su Park, Dong-Il Sun, Joo Yeon Jhun, Hye Jwa Oh, Sung-Hwan Park, Ho-Youn Kim

**Affiliations:** 1 Division of Rheumatology, Department of Internal Medicine, Chungbuk National University Hospital, 410, Gaesin-dong, Heungduk-gu, Cheongju, 361-711, South Korea; 2 Division of Rheumatology, Department of Internal Medicine, Gyongsang University, 90, Chilam-dong, Jinju, 660-702, South Korea; 3 Division of Rheumatology, Department of Internal Medicine, College of Medicine, The Catholic University of Korea, 505, Banpo-Dong, Seocho-Gu, Seoul, 137-701, South Korea; 4 Department of Otolaryngology-Head and Neck Surgery, College of Medicine, The Catholic University of Korea, 505, Banpo-Dong, Seocho-Gu, Seoul, 137-701, South Korea; 5 Rheumatism Research Center, Catholic Institutes of Medical Science, The Catholic University of Korea, 505, Banpo-Dong, Seocho-Gu, Seoul, 137-701, South Korea

**Keywords:** IL-21, IL-21 receptor, Sjogren's syndrome, Immunoglobulin G1, Labial salivary gland

## Abstract

**Introduction:**

Interleukin (IL)-21 is a cytokine that controls the functional activity of effector T helper cells and the differentiation of Th17 cells, and promotes B-cell differentiation. To test whether IL-21 participates in the pathogenesis of primary Sjögren's syndrome (SS), serum IL-21 level was measured and IL-21 expression in the labial salivary glands (LSG) was examined.

**Methods:**

Serum IL-21 levels in 40 primary SS, 40 rheumatoid arthritis (RA), and 38 systemic lupus erythematosus (SLE) patients and 20 healthy controls were measured. Serum IL-21 levels of SS patients were assessed for correlations with laboratory data, including anti-nuclear antibody, anti-Ro/La antibodies, globulin, immunoglobulin (Ig) class, and IgG subclass. LSGs from 16 primary SS and 4 controls with sicca symptoms were evaluated for IL-21 and IL-21 receptor (IL-21R) expression by immunohistochemistry. Confocal microscopy was performed to further characterize the IL-21 positive cells.

**Results:**

Primary SS patients had significantly higher serum IL-21 levels than controls, and these increments correlated positively with levels of IgG, IgG1. Serum IgG1 levels correlated with anti-Ro antibody titers. Immunohistochemical analyses showed that lymphocytic foci and the periductal area of the LSGs from SS patients expressed high levels of IL-21 and lower levels of IL-21R, whereas the control LSGs showed minimal expression of both antigens. The more the lymphocyte infiltrated, IL-21expression in LSGs showed a tendency to increase. Confocal microscopic analyses revealed that IL-21 expressing infiltrating lymphocytes in the LSGs of SS patients also expressed CXCR5.

**Conclusions:**

Primary SS is associated with high serum IL-21 levels that correlate positively with serum IgG, especially IgG1, levels. The expression of IL-21 is increased as more lymphocytes infiltrated in LSGs. These observations suggest that IL-21 may play an important role in primary SS pathogenesis.

## Introduction

IL-21 is a pleiotropic cytokine that belongs to the common cytokine receptor γ chain (γc)-dependent cytokine family, which is produced by activated CD4+ T cells and NKT cells [1]. The IL-21 receptor (IL-21R) consists of the IL-21R α chain and the γc chain and is expressed on T cells, NK cells, NKT cells, B cells, dendritic cells (DCs) and macrophages as well as on non-hematopoietic cells, including keratinocytes and fibroblasts [2]. The activation of IL-21R by the binding of IL-21 enhances the proliferation of T cells after their prior stimulation with anti-CD3 [3]. IL-21 also controls the functional activity of effector T helper (Th) cells and the differentiation of Th17 cells, and counteracts the suppressive effects of regulatory T cells [4]. IL-21 alone is capable of directly inducing both B lymphocyte-induced maturation protein-1 (Blimp-1), which is required for plasma-cell differentiation, and Bcl-6, which is required for germinal center reactions [5]. IL-21 also promotes B-cell differentiation by synergizing with BAFF and enhancing the CD40-mediated induction of activation-induced deaminase (AID) and Blimp1 [6]. Overexpression of IL-21 in mice results in hypergammaglobulinemia and autoantibody production [7].

Primary Sjögren's syndrome (SS) is a systemic autoimmune disease characterized by keratoconjunctivitis sicca, xerostomia, and extraglandular abnormalities [8]. At the immunological level, it is characterized by both T-cell lymphocytic infiltration of the exocrine glands and B-cell hyper-reactivity. Hypergammaglobulinemia is a common laboratory finding in primary SS. Since previous observations suggest that IL-21 and IL-21R may be associated with immunoglobulin production, autoantibody production, and B-lymphocyte hyperactivity [3,9], it is thought that IL-21 is involved in the pathogenesis of autoimmune disease. However, the precise roles IL-21 and IL-21R in human autoimmune disease are still poorly understood. To determine whether IL-21 participates in primary SS pathogenesis, we examined whether the serum IL-21 levels of patients with primary SS correlate with various laboratory parameters. The expression of the IL-21/IL-21R cytokine/receptor pair by the salivary glands of patients with primary SS was also investigated.

## Materials and methods

### Patients and samples

The participants were selected from patients at Seoul Saint Mary's hospital at the Catholic University of Korea. Written informed consent was obtained from all patients and controls. The study was approved by the ethical committee of the Seoul St. Mary's Hospital (KC09FZZZ0522). For serological studies, serum samples were obtained from 40 patients with primary SS, 38 patients with systemic lupus erythematosus (SLE), 40 patients with rheumatoid arthritis (RA), and 20 healthy controls. All SLE and RA patients fulfilled American College of Rheumatology (ACR) classification criteria [10,11]. The consent form was approved by the Hospital Ethics Committee. In addition, labial salivary gland (LSG) biopsy specimens were collected from 16 patients (all women) that matched the histological criteria for a diagnosis of SS [12] and had severe cellular infiltration (focus score ≥1). All patients were female. The mean age and the duration of disease were 51.7 ± 9.5 years and 0.3 ± 1.0 years. The biopsies were performed for routine diagnostic purposes after obtaining the patient's consent. All patients diagnosed with primary SS fulfilled the American-European Consensus Group Criteria for this diagnosis [13]. There were also four control LSG specimens from subjects who did not fulfill the classification criteria for primary SS but had sicca symptoms, such as dry mouth or dry eyes. The controls were matched for sex and age to the primary SS patients and had been examined for the presence of rheumatic disease, including secondary SS.

### Clinical and immunological data

All patients underwent extensive medical examinations and serological evaluations, including measurements of anti-nuclear antibody (ANA), rheumatoid factor (RF), globulins and Ig classes, along with the erythrocyte sedimentation rate (ESR). In addition, the anti-Ro/SSA, anti-La/SSB, and IgG subclass titers in primary SS patients were measured.

The anti-Ro/SSA and anti-La/SSB antibodies were tested by using a commercially available enzyme-linked immunosorbent assay (ELISA) kit (BIO QUANT, San Diego, CA and USA). To measure IgG1, IgG2, IgG3 and IgG4 titers, a Human IgG Subclass Profile ELISA kit (Invitrogen, Camarillo, CA, USA) was used.

### Labial salivary gland biopsy

LSG biopsies were taken from primary SS patients at an otorhinolaryngology clinic. For this, local anesthetic was injected into the lower lip and a small incision to the right or left of the lip midline was made. Five or six LSG lobules were harvested carefully and placed into 10% phosphate-buffered formalin for 24 hours. Standard paraffin preparations were prepared, sectioned at a thickness of 5 μm, and stained with hematoxylin and eosin. The slides were examined for the presence of lymphocytic infiltrates and/or foci by three observers using standardized criteria. The focus score was reported as the number of foci per 4 mm^2 ^of tissue.

### Measurement of serum IL-21

The IL-21 concentrations in the sera from the 40 primary SS patients, 38 SLE patients, 40 RA patients, and 20 healthy controls were measured by sandwich ELISA. Briefly, monoclonal capture antibodies (eBioscience, San Diego, CA and USA) were added to a 96-well plate (Nunc) and incubated overnight at 4°C, after which the plates were washed five times with PBS containing 0.05% Tween20. Following incubation with blocking solution for one hour at room temperature, the test samples and recombinant IL-21 (eBioscience) standards were added to the plates. The plates were incubated for two hours at room temperature, after which they were washed five times. Biotin-conjugated anti-human IL-21 antibodies (eBioscience) were added and, incubated for an hour at room temperature and the plates were washed. Avidin-horseradish peroxidase (eBioscience) was added and the reaction was allowed to proceed for 30 minutes at room temperature. The plates were then washed five times, and TMB solution was added to induce the color reaction, which was stopped by adding 2N H_2_SO_4_. The optical density at 450 nm was measured by using an automated microplate reader (VERSAmax, Molecular Devices, Palo Alto, CA, USA). A standard curve was drawn by plotting the optical density against the log of the concentration of IL-21.

### Immunohistochemical staining for IL-21 and IL-21R

The fixed LSG biopsy specimen slides were deparaffinized by immersion in xylene, followed by dehydration in ethanol. The sections were incubated for 30 minutes at room temperature with blocking solution containing normal sera and avidin block (Vector Laboratories, Burlingame, CA, USA), and then incubated overnight at 4°C with either anti-IL-21 (Santa Cruz Technology Inc., Santa Cruz, CA, USA) or anti-IL-21R, both of which were diluted 1:100 (R&D Systems, Minneapolis, MN and USA). Mouse IgG or goat IgG served as isotype controls. The slides were washed for 5 minutes, followed by a 20-minute incubation with biotinylated anti-rabbit IgG or biotinylated anti-goat IgG (Vector). Following a 15-minute wash, the slides were incubated for 1 h with horseradish peroxidase-conjugated avidin-biotin by using the Vectastain ABC Elite (Vector). The stain was developed by using diaminobenzidine substrate (DAKO, Carpinteria, CA, USA). Counterstaining was performed with hematoxylin. Enumeration of IL-21-positive cells was performed in the vicinity of LSG lymphocytic infiltration and in the interstitium. One lymphocytic focus per specimen was randomly selected for enumerating the number of positively stained cells. Quantification of IL-21-positive cells was performed with the HistoQuest analysis software (TissueGnostics, Los Angeles, CA and USA).

### Confocal microscopic analysis of LSG co-expression of CD4, CD20, CXCR5, and IL-21

Salivary gland pieces from eight patients were analyzed. Cryosections (7-μm thick) were fixed with acetone, blocked with 10% goat serum, and stained with anti-CD4-FITC, anti-CD20-FITC (BD Biosciences, Franklin Lakes, NJ and USA), anti-CXCR5-PerCP-Cy5.5 (Biolegend, San Diego, CA and USA), and anti-IL-21-PE (eBioscience). Fluorescence images were acquired by using a LSM 510 confocal microscope (Zeiss, Berlin, Brandenburg and Germany).

### Statistical analysis

Statistical analyses were performed by using SAS software (Version 9) (Cary, North Carolina and USA). The data were expressed as individual values with median. Differences in the mean IL-21 levels of various groups were analyzed by using the Kruskal-Wallis test. Differences between the two groups were analyzed by using the Mann-Whitney test (two-tailed). Spearman's rank test was used to assess the correlation between the levels of IL-21 and laboratory data. Values of *P *< 0.05 were considered significant.

## Results

### Primary SS patients have higher serum IL-21 levels than healthy controls

**Figure 1:**
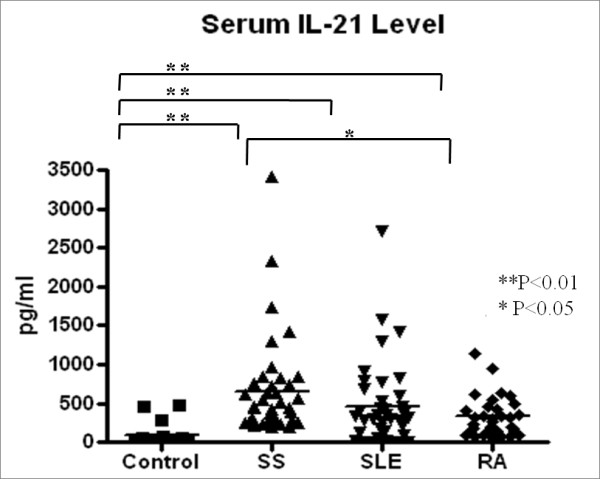
**The serum IL-21 level**. The serum IL-21 levels of patients with primary SS (*n *= 40), SLE (*n *= 38), or RA (*n *= 40) and healthy controls (*n *= 20) were measured by ELISA. The primary SS patients had significantly higher serum IL-21 levels than the healthy controls or the RA patients. Horizontal bars represent the median of all samples within a group. * *P *< 0.05, ** *P *< 0.01. IL: Interleukin; RA: rheumatoid arthritis; SLE: systemic lupus erythematosus; SS: Sjögren's syndrome.

Measurement of the serum IL-21 levels in primary SS (*n *= 40), SLE (*n *= 38), and RA (*n *= 40) patients and healthy controls (*n *= 20) by ELISA revealed mean IL-21 levels of 646 ± 637, 454 ± 534, 342 ± 322, and 74 ± 132 pg/ml, respectively (Figure 1). The serum IL-21 levels varied significantly among patients with primary SS, SLE, RA and controls (*P *< 0.01). The levels of primary SS group was notably higher than the control levels (*P *< 0.01). The primary SS group also had higher IL-21 levels than the RA group (*P *< 0.05) but did not differ significantly from the SLE group.

### The serum IL-21 levels in patients with primary SS correlate with their globulin, IgG, and IgG1 levels

**Figure 2:**
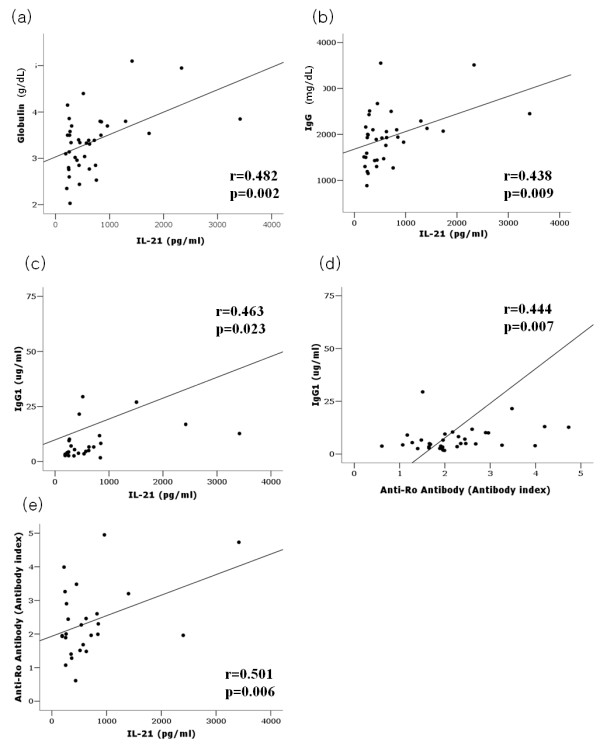
**Correlations between the serum IL-21 levels of primary SS patients and the levels of immunoglobulin**. **(a) **The serum IL-21 levels of primary SS patients (*n *= 40) correlated significantly with the serum globulin levels. **(b) **Of all the Ig classes, the serum IL-21 levels only correlated with IgG (*n *= 40). **(c) **There was a positive correlation between the serum IL-21 and IgG1 levels (*n *= 24). **(d) **The anti-Ro/SSA antibody titers, as measured by ELISA, correlated with the IgG1 levels (*n *= 35). **(e) **The serum IL-21 levels correlated with anti-Ro/SSA antibody index (*n *= 24) and IL-21 was an independent variable in regression analysis. The correlation coefficients (r) and *P-*alues of statistical significance are shown. IG: immunoglobulin; IL: Interleukin; SS: Sjögren's syndrome.

To identify possible correlations between serum IL-21 levels and laboratory data of primary SS, the clinical data of the 40 primary SS patients were examined. The patients had normal clinical test results, except for the tests measuring the levels of ANA, anti-Ro/SSA, anti-La/SSB, RF, globulin, and the ESR. The serum IL-21 levels of the patients did not correlate with ANA or RF titers or ESR, but did correlate with globulin and IgG levels (globulin: γ = 0.482, *P *= 0.002; IgG: γ = 0.438, *P *= 0.009). The regression lines for these correlations are shown by the lines in Figures 2a, b. On the other hand, the IL-21 levels did not correlate significantly with other Ig classes. Among IgG subclasses, only the IgG1 levels correlated significantly with IL-21 levels (r = 0.463, *P *= 0.023), as shown in Figure 2c. Figure 2d shows that the IgG1 levels also correlated with the anti-Ro/SSA antibody titers (r = 0.444, *P *= 0.007). IL-21 also correlated with anti-Ro/SSA antibody titers (Figure 2e) and IL-21 was an independent variable explaining the anti-Ro/SSA antibody in regression analysis, as shown in Table 1 (r = 0.501, *P *= 0.006). The patients with and without extra-glandular manifestations did not differ significantly in terms of serum IL-21 levels (data not shown).

**Table 1:** Multivariate regression analysis between IL-21, IgG1 and Anti-Ro Antibody

	Anti-Ro antibody			
	**B**	SE*	**95% CI**	***P*-value**
**Constant**	1.709	0.236	1.218 to 2.200	0.000
**IL-21 (pg/ml)**	0.001	0.000	0.000 to 0.001	0.014
**IgG1 (ug/ml)**	0.013	0.004	0.004 to 0.022	0.008

*SE: standard error.

CI: confidence interval; Ig: immunoglobin; IL: interleukin.

### Expression of IL-21 and IL-21R in LSGs from patients with primary SS

**Figure 3:**
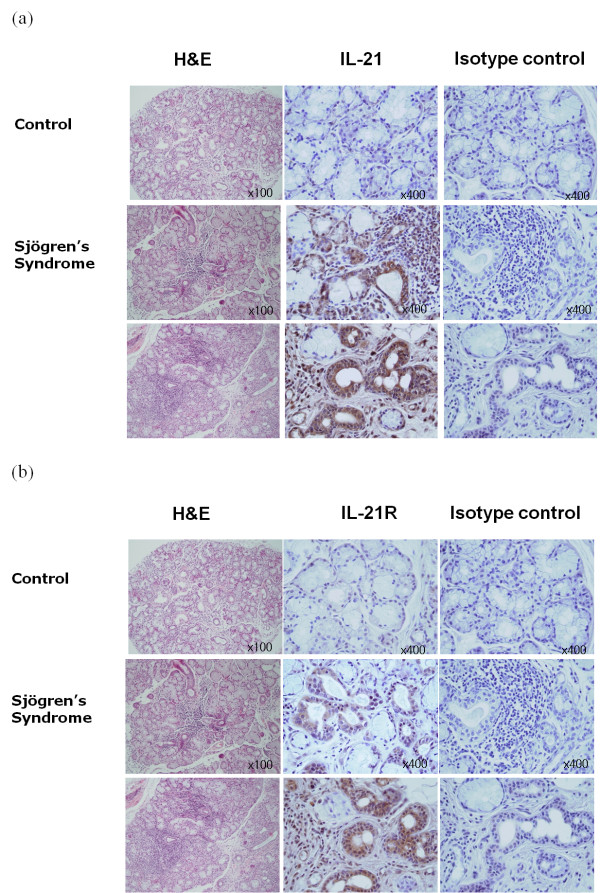
**The labial salivary glands (LSG) of primary SS exhibit increased IL-21 and IL-21 receptor expression**. Shown is the expression of the IL-21 and IL-21 receptor in the labial salivary glands of control subjects (*n *= 4; upper panel) and patients with primary SS (*n *= 16; lower two panels), as determined by immunostaining using specific antibodies. The cells that stained with the antibodies appeared in brown. **(a) **The infiltrating lymphocytes and periductal areas of the patients with SS exhibited intense IL-21 staining, whereas there was no staining with the isotype controls. **(b) **The lymphocytic infiltrations and periductal areas stained for IL-21 receptor. In contrast, the control subjects did not exhibit any IL-21 or IL-21 receptor expression in their labial salivary glands. IL: Interleukin; LSGs: labial salivary glands; SS: Sjögren's syndrome.

**Figure 4:**
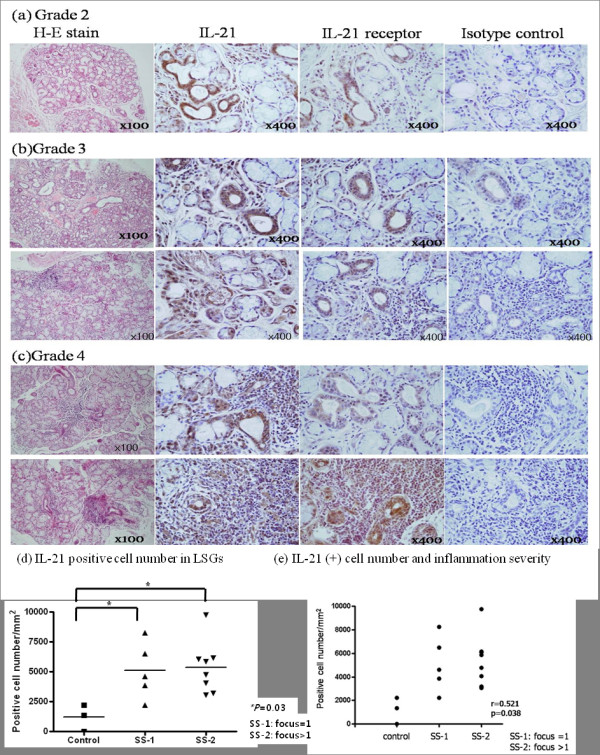
**The expression of IL-21 in the LSG of SS patients and the severity of inflammation**. The labial salivary gland specimens of the primary SS patients were divided according to the grade (1 to 4) of lymphocytic infiltration (see Methods). **(a) **This specimen was obtained from a SS patient who fulfilled the American-European Consensus Group Criteria except LSG biopsy. There were no definite lymphocytic foci but moderate lymphocytic infiltration was observed. **(a-c) **IL-21 expression increased as the lymphocytic infiltration in the salivary gland became more severe. **(d) **IL-21 positive cell number was significantly increased in LSG from SS patients than in control. **(e) **Relationship between IL-21 positive cell number and inflammation severity. IL-21 positive cell number was elavated with grade (r = 0.521, *P *= 0.038). Horizontal bars represent the median of all samples within a group. Representative data are shown. IL-21: Interleukin-21; LSGs: labial salivary glands; SS: Sjögren's syndrome.

Immunohistochemical analyses were performed to determine whether IL-21 and IL-21R are expressed by the LSGs of 16 primary SS patients and 4 healthy controls. The freshly explanted lower lip biopsy specimens were sectioned and stained with anti-IL-21 and anti-IL-21R antibodies. All 16 primary SS samples exhibited distinct expression of IL-21 (Figure 3a), while 14 of the 16 samples were positive for IL-21R (Figure 3b). In contrast, none of the LSGs from the healthy controls exhibited IL-21 and IL-21R expression (Figure 4). The 14 primary SS patients stained for IL-21R showed similar patterns of IL-21 and IL-21R expression; that is, IL-21 was prominent in infiltrating lymphocytes and ductal cells, while IL-21R was mainly expressed on ductal cells. A weak expression of IL-21R was also observed in lymphocytic infiltrates. Staining of both IL-21 and IL-21R appeared as a diffuse pattern. Overall, the expression of IL-21 was stronger and more widely distributed than IL-21R. Both IL-21 and IL-21R were expressed on lymphocytic infiltrates and to a lesser degree in the acinar components (Figure 3).

### Immunohistochemical analysis of IL-21 and IL-21R

The primary SS patients were divided according to their grade of lymphocytic infiltration (grades 1 to 4; described in the Methods). A grade 2 specimen is shown in Figure 4a; this specimen was obtained from a primary SS patient who fulfilled the American-European Consensus Group Criteria, except LSG biopsy. Moderate lymphocytic infiltration was observed but a definite lymphocytic focus was not seen. The specimens that had grade 3 or 4 had one or more foci. IL-21 positive cell number was increased more in both grade 3 and grade 4 than in the control, as shown in Figure 4d. Though there are no significant differences between grade 3 and grade 4, the more severe the lymphocytic infiltration was, the more IL-21 was expressed on both the infiltrating lymphocytes and periductal area (Figure 4e). However, we could not show a significant relationship between IL-21R positive cell number and the severity of lymphocytic infiltration.

### Co-expression of IL-21 and CXCR5 in the LSGs of patients with primary SS

**Figure 5:**
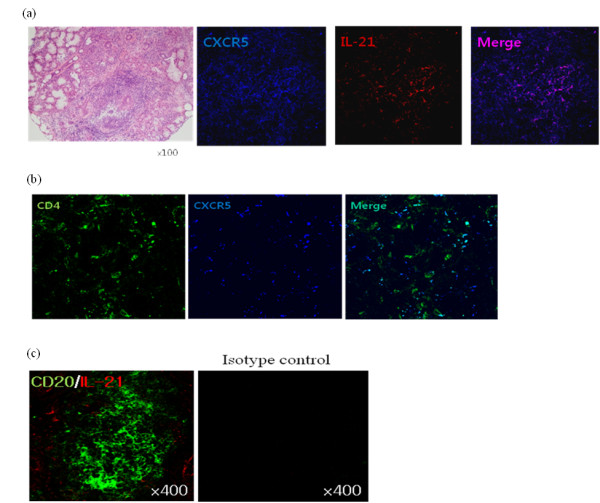
**Confocal microscopic analysis of the IL-21-expressing cells in the LSGs of primary SS patients**. **(a) **Immunofluorescent double-staining of IL-21-expressing cells (red) and CXCR5+ cells (blue). **(b) **Immunofluorescent double-staining of CD4+ T cells (green) and CXCR5+ cells (blue). **(c) **Immunofluorescent double-staining of IL-21-expressing cells (red) and CD20+ B cells (green). Only the IL-21-expressing cells merged with the CXCR5+ cells. IL-21: Interleukin-21; LSGs: labial salivary glands; SS: Sjögren's syndrome.

Earlier studies have reported that the LSGs of primary SS patients can have a germinal center-like structure that is composed of follicular helper T cells (TfH), B cells and DCs [14,15]. It is also known that TfH cells produce IL-21, which promotes the expression of the TfH marker CXCR5 [16]. We investigated whether LSG expression of IL-21 corresponds to its CXCR5 expression. Indeed, confocal microscopy revealed that the IL-21 expression of LSGs from primary SS patients coincided with their CXCR5 expression (Figure 5a). Since B cells are also known to express CXCR5 [17], we assessed whether IL-21 co-expressed with B cells as well. However, IL-21 expression did not merge with the expression of CD20, a B-cell marker (Figure 5c).

## Discussion

In this study, we assessed the expression pattern of IL-21 and IL-21R in LSG tissues of primary SS patients. Our results encouraged the original observation demonstrating the correlation between IL-21 expression and IgG levels in association with disease severity of SS.

The role of IL-21 in the pathogenesis of SS is poorly understood, but several lines of thought support the notion that it may contribute to a pivotal role in the process of disease. First, it is known that IL-21 is involved in the proliferation and survival of B cells, promoting their differentiation into Ig-producing plasma cells [1]. The latter ability of IL-21 [7,18] suggests that this cytokine may play important roles in B cell-mediated autoimmune diseases and allergies. Second, naïve IL-21R KO mice have diminished serum IgG1 levels and immunization of these mice with T-dependent antigen results in lower antigen-specific IgG1 levels compared to wild-type mice [3]. Third, SS patients have increased IgG levels in their serum, although the reason for this increment has not been explained. The B-cell infiltration in the LSGs from primary SS patients is less than the T-cell infiltration; however, B-cell hyperactivity is an important characteristic of primary SS. Indeed, a prominent humoral autoimmune response in patients with primary SS is the production of anti-Ro/SSA antibodies. Moreover, with regard to the IgG subclasses of the anti-Ro antibodies, IgG1 predominates [19]. In this study, we found that the serum IL-21 levels correlated with the serum globulin, IgG, and, in particular, IgG1 levels in primary SS patients. These observations are consistent with the role IL-21 plays in plasma-cell differentiation [7]. In addition, we found the IgG1 levels correlated with the serum anti-Ro/SSA antibody titers. Thus, our observations support the notion that IL-21 may promote autoantibody production by inducing IgG class switch recombination in B cells.

IL-21 regulates the activation, proliferation and survival of CD4+ T cells as well as that of B cells [20,21]. Specifically, it up-regulates the expression of the ROR γt transcription factor, promotes Th17-cell differentiation, and induces IL-23R expression on Th17 cells, thus facilitating the ability of IL-23 to expand the Th17-cell response [22,23]. IL-21 also regulates the functional activity of CD8+ T cells and NK cells [20,21]. Naive CD8+ T cells express low levels of IL-21R [24] and, although IL-21 does not by itself induce CD8+ T-cell proliferation, it acts synergistically with IL-15 or IL-7 to induce the proliferation of both naïve and memory phenotype CD8+ T cells [25].

Our study showed that IL-21 correlates with the degree of lymphocytic infiltration in the LSGs of patients with primary SS. The lymphocytic foci and periductal areas of the LSGs had many IL-21 and IL-21R positive cells. In contrast, the LSGs of healthy control subjects did not express IL-21 or IL-21R. Together these data suggest that IL-21 and IL-21Rpositive cells may play an important role in the sialoadenitis suffered by patients with primary SS.

Early studies showing that two mouse models of SLE have elevated IL-21 levels suggested that IL-21 may play a role in autoimmunity. Herber *et al*. showed that, in the MRL-Fas^lpr ^mouse model of SLE, blocking IL-21 with IL-21R-Fc reduced renal disease lymphadenopathy, skin lesions, and circulating autoantibodies and IgG [26]. Similarly, administration of an IL-21R-Fc fusion protein to BXSB.B6-Yaa+/J mice, the second SLE animal model, decreased IL-21 production, lymphocyte activation and circulating IgG1 levels [27]. These data are in agreement with the results from our study, suggesting the notion that IL-21 may participate in autoimmune disease pathogenesis by influencing lymphocytic activation and IgG production. Further supporting this possibility is that the blockade of endogenous IL-21 activity by an IL-21R-Fc fusion protein in the cultured RA synovial membrane cells significantly inhibited the production of inflammatory cytokines [28]. Moreover, administration of IL-21R-Fc to collagen-induced arthritis mice and adjuvant-induced arthritis rats alleviated their clinical and histological signs of inflammation [9].

Additional lines of evidence also suggest that IL-21 may promote human autoimmune disease in general. Yuan *et al*. have reported that patients with SS have higher serum IL-21 levels than healthy control subjects, which correlated positively with gamma-globulin levels [29]. Moreover, by using a large sample of SLE patients and healthy controls, Sawalha *et al*. found that SLE is associated with two single nucleotide polymorphisms of the IL-21 gene [30]. In addition, patients with SLE have elevated IL-21 serum levels that correlate with the severity of the disease [31]. Similarly, Astrid *et al*. have reported that synovial fibroblasts and synovial macrophages of patients with RA express IL-21R (IL-21 was not tested) [32]. In addition, the epidermis of patients with systemic sclerosis expressed IL-21R (but not IL-21) [33]. Recently, genome-wide association studies have provided convincing evidence that the chromosomal 4g 27 region that harbors the IL-21 and IL-21 genes is associated with chronic inflammatory disorders, including SLE, inflammatory bowel disease and psoriasis [30,34,35].

Interestingly, two previous studies reported that they were not able to detect IL-21 in the synovium of RA patients or the skin of systemic sclerosis patients [32,33]. This may relate to an observation made in the experimental autoimmune encephalitis (EAE) animal model; while the treatment of mice with IL-21 after EAE was induced did not affect the severity of the disease, treatment before disease induction greatly enhanced the inflammatory influx into the central nervous system and the severity of the disease [36]. The latter mice also exhibited higher levels of circulating myelin-specific antibodies and IFN-γ, but the level of IL-4 remained similar to that of control mice. Thus, the effects of IL-21 may be specific to the early stages of the autoimmune response. We found what may support this hypothesis. When we measured the serum levels of proinflammatory cytokines, such as IL-6, IL-17 and IL-23, to determine whether serum IL-21 expression correlates with systemic inflammation, we failed to detect any significant correlations (data not shown). Thus, it is possible that IL-21 is involved at the onset of autoimmune disease rather than later during the active systemic inflammation stage. In the present study, the LSG biopsies were performed for diagnostic purposes, thus the biopsy specimens are likely to be tissues from the early phase of primary SS. Since we found that IL-21 and IL-21R are expressed in these LSGs, it may be that IL-21 plays an important role in the onset and early development of primary SS.

Our data showed that IL-21 expression was prominent in infiltrating lymphocytes and ductal cells. It is not yet known whether the epithelial cells lining the duct produce IL-21. Therefore, we could not completely exclude the possibility that the high expression of IL-21 in the ductal cell is the specificity of the reagents or the artifact in the staining procedure. However, it is known that IL-21 is expressed in non-immune cells as well as in lymphocyte. IL-21 expression is enhanced at the site of the involved gut in Crohn's disease [37]. IL-21 is also expressed in neurons in the gray matter in multiple sclerosis [38]. This suggested the possibility that IL-21 can be produced in non-immune cells, including epithelial cells.

The glandular destruction in SS has been shown to be mediated mainly by primed CD4+ T cells [39]. Of the lymphocytes that infiltrate the salivary glands in patients with SS, 45 to 50% are CD4+ T cells, 20% are CD8+ T cells, and about 20% are B cells [40]. Interestingly, the confocal microscopic analyses in the present study revealed that the LSG-infiltrating cells that stained for IL-21 also stained for CXCR5. CXCR5 is a chemokine receptor that is expressed by all mature B cells as well as by a subset of antigen-experienced CD4+ T cells in lymphoid tissue [41]. CXCR5 is strongly implicated in the follicular migration of early-activated T cells and their consequent co-localization with B cells [42]. The priming of CD4+ T cells in lymphoid tissue result in their novel expression of CXCR5 [43,44]. However, CXCR5 is rapidly lost during T-cell proliferation, which indicates that the follicular homing program is an early and transient stage in the T-cell activation and differentiation process [44,45]. Continued CXCR5 expression after priming may reflect qualitative or quantitative aspects of this stimulation [46]. Since the majority of the T cells in the salivary glands of primary SS patients are CD4+ T cells, and the IL-21 produced by activated CD4+ T cells is known to up-regulate their expression of CXCR5 [16], IL-21 possibly promotes the co-localization of T cells and B cells in the functional ectopic germinal centers in the salivary glands of SS patients, which in turn elevates autoantibody production. Taken together, these observations suggest that high expression of IL-21 may play an important role in the pathogenesis of primary SS by affecting both T cells and B cells.

## Conclusions

Our present study is the first to report that there is a relationship between the elevated serum levels of IL-21 and IgG, (especially IgG1), in primary SS and that the lymphocytic infiltration in the LSGs of patients with primary SS express high levels of IL-21 along with lower levels of IL-21R. Moreover, the expression of IL-21 showed a tendency to increase as more lymphocytes infiltrated into LSGs. Further investigations on the systemic and localized effects of IL-21 in primary SS would provide a basis for targeting this molecule for customized therapy.

## Abbreviations

AID: activation-induced deaminase; ANA: anti-nuclear antibody; Blimp-1: B lymphocyte-induced maturation protein-1; EAE: experimental autoimmune encephalitis; ELIZA: enzyme-linked immunosorbent assay; ESR: erythrocyte sedimentation rate; IG: immunoglobulin; IL-21: Interleukin-21; IL-21R: Interleukin-21 receptor; LSGs: labial salivary glands; RA: rheumatoid arthritis; RF: rheumatoid factor; SLE: systemic lupus erythematosus; SS: Sjögren's syndrome; TfH: follicular helper T cells; Th cells: T helper cells.

## Competing interests

The authors declare that they have no competing interests.

## Authors' contributions

KYK contributed to conception and design, acquisition of data, analysis and interpretation of data, and drafting of the article. HOK performed statistical analysis and acquisition of data. SKK contributed to the interpretation of the study and revised the manuscript critically for important intellectual content. JHJ contributed to design and statistical analysis, and drafted the manuscript. KSP participated in the study's conception and coordination and drafted the manuscript. DIS contributed to acquisition of data and Immunohistochemical staining. JYJ carried out the ELISA and drafted the manuscript. HJO carried out Immunohistochemical staining and drafted the manuscript. SHP contributed to conception, design, acquisition of data, and helped to draft the manuscript. HYK contributed to design, acquisition of data, analysis and interpretation of data, and revision of the article. All authors read and approved the final manuscript for publication.
